# Pneumonia severity index in viral community acquired pneumonia in adults

**DOI:** 10.1371/journal.pone.0210102

**Published:** 2019-03-06

**Authors:** Mi-Ae Kim, Jae Seok Park, Choong Won Lee, Won-Il Choi

**Affiliations:** 1 Department of Internal Medicine, Keimyung University Dongsan Hospital, Daegu, Republic of Korea; 2 Department of Occupational & Environmental Medicine, Sungso Hospital, Andong, Republic of Korea; Kliniken der Stadt Köln gGmbH, GERMANY

## Abstract

Pneumonia severity index (PSI) is an important scoring system that can assess the severity of community acquired pneumonia and determine admission status. However, there is a lack of research on whether this scoring system can be applied to viral community acquired pneumonia. The purpose of this study was to evaluate the usefulness of PSI in viral community acquired pneumonia. This retrospective cohort study included 1,434 adult patients (aged ≥18 years) who were admitted to the emergency department of a university hospital during 2013–2015 because of community-acquired pneumonia. Viral infections were diagnosed by multiplex PCR. Patients diagnosed with non-viral community-acquired pneumonia were included in the control group (N = 1,173). The main outcome was 30-day all-cause mortality. multivariate Cox regression analyses were performed to calculate the risk of death. Respiratory viruses were detected in 261 (18.2%) patients with community-acquired pneumonia. Two types of respiratory viruses were detected in 7 cases. Of the 254 cases detected with only one virus, 62 were influenza A, 18 were influenza B, 65 were rhinovirus, 35 were respiratory syncytial virus, 25 were metapneumovirus, 20 were parainfluenza, 17 were coronavirus, 7 were bocavirus, and 5 were adenovirus. Mortality was not significantly different between patients with respiratory virus and those without respiratory virus; the 30-day all-cause mortality rates were 20.3% and 22.4%, respectively (P = 0.45). Mortality rate increased with an increasing PSI score with or without respiratory viral infection. Pulmonary severity index was significantly associated with mortality adjusted for respiratory virus detection (hazard ratio = 1.024, 95% confidence interval = 1.020–1.028). Pneumonia severity index score is an important factor for assessing the prognosis of patients with community-acquired pneumonia, regardless of respiratory virus detection.

## Introduction

Viruses are a frequent cause of community associated pneumonia (CAP) [1]. Among CAP, outpatient treatment mortality ranges from 1% to 5%, but mortality rates in hospitalized patients are about 12% [2]. Based on these mortality rates, a tool for predicting the severity of CAP has been developed and is actively used in clinical practice [3, 4]. However, mortality of viral CAP has not been well validated by the pneumonia severity index (PSI).

It is well known that the prognosis of community acquired pulmonary disease is influenced by the pathogen itself. However, co-morbidities of the patient and severity of infection at the time of diagnosis have been regarded as important factors in prognosis [3, 4]. In adults, viral CAP has a reported mortality from 1% to 39% [5–11]. The mortality rate of viral CAP was lower in patients with outpatient treatment, but increased with patient age [5, 8–10]. Unlike pediatric patients, many adult patients have multiple co-morbidities. In adult patients with viral CAP, coexisting disease, severity of infection at diagnosis, and age, rather than causative organism, have a significant impact on prognosis [3, 4]. However, the mortality of viral CAP has not been well considered with co-founders or co-morbidities in adults.

Since the introduction of highly sensitive viral detection methods such as polymerase chain reaction (PCR), the incidence of respiratory viruses has been increasing in community-acquired pneumonia [12]. The development of the PCR method has made it possible to simultaneously diagnose multiple respiratory viruses using one sample [1, 13, 14]. The development and introduction of sensitive testing methods will require periodic confirmation of viral CAP epidemiology, and adjustment of its mortality rate and established prognostic factors.

We aimed to investigate the distribution pneumonia severity index (PSI) class and whether PSI play a role in prognostic factors in viral CAP. We studied the 30-day all-cause mortality rate for hospital admitted CAP patients with respiratory viruses in relation to pneumonia severity index (PSI).

## Methods

### Study population

**Fig 1:**
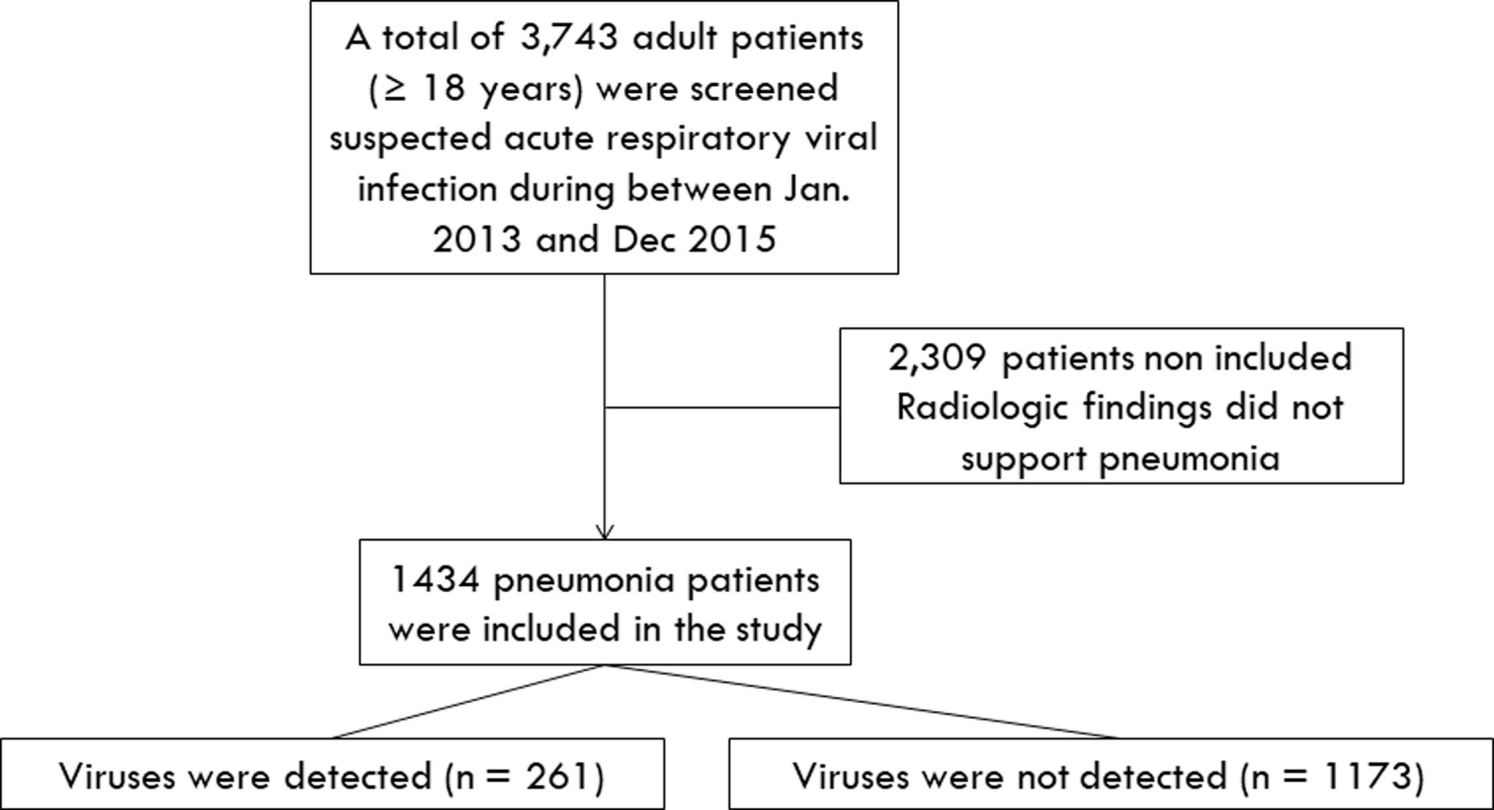
Flow chart of patients who had pneumonia.

A retrospective cohort study of hospitalized adults CAP patients with respiratory virus infection was conducted. All patients aged ≥18 years admitted between January 2013 and December 2015, with suspected respiratory viral infections (N = 3,743), were studied. If physicians suspected respiratory viral illness, then virus multiplex PCR was performed. We excluded 2,309 subjects who had no identifiable radiologic pneumonia findings (Fig 1). Viruses detected CAP patients (n = 261) were compared with patients diagnosed with viruses not detected CAP (n = 1173) during the same period (2013–2015). Two patients had a mixed infection of bocavirus and parainfluenza. Among RSV-infected patients, one had mixed infection with rhinovirus and one patient had influenza. Among influenza-infected patients, two patients had mixed infection with bocavirus. All the patients with mixed infections were included in this study.

The current study was approved by the institutional review board (IRB) at Dongsan Hospital, Keimyung University School of Medicine. The IRB waived the requirement for informed consent. This study was conducted in compliance with the Declaration of Helsinki.

### Definitions

Pneumonia was defined as the presence of a new or progressive infiltrate found using either chest radiography or chest CT scan, in addition to two or more of the following: fever, sputum production, rhinorrhea, sore throat, dyspnea, or a diagnosis of pneumonia by the attending physician. The outcome was designated as all-cause mortality up to 30 days after hospital admission.

### Specimens

During the study period, nasopharyngeal specimens were obtained using flocked swabs and stored and transported using the universal transport medium (Nobelbio, Hwaseong, Korea). Nasopharyngeal specimens were submitted for respiratory virus detection. Nucleic acids were extracted from 300μL specimens using a Viral Gene-spin^TM^ Viral DNA/RNA Extraction Kit (iNtRON Biotechnology, Seongnam, Korea). cDNA was synthesized from each of the extracted RNA samples with cDNA Synthesis Premix (Seegene, KySeoul, Korea) and a GeneAmp PCR System 9700 thermal cycler (Applied Biomaterials, Foster City, CA, USA). Blood culture was performed at every pneumonia patient. Sputum culture was performed if patients expectorated sputum.

### Respiratory virus testing

Respiratory virus (RV) 13 testing was performed to detect the following viruses: adenovirus(A~F), influenza viruses A and B, RSV A, RSV B, parainfluenza viruses 1 to 3, Rhinovirus, metapneumovirus, coronavirus 229E, coronavirus OC43, and bocavirus. During the RV13 test, an internal control was added to each specimen to check the entire process from nucleic acid extraction to PCR, according to the manufacturer's instructions. An Anyplex II RV13 Detection Kit (BioSewoom, Seoul, Korea) was used, according to the manufacturer's instructions.

### Data collection

This study was performed at Keimyung University Dongsan Hospital, a 867-bed, tertiary care teaching hospital in Daegu, Republic of Korea. If a patient had an episode of acute respiratory infection at an emergency department or outpatient clinic or within 2 days during admission, he or she underwent multiplex RT-PCR testing. Adult patients (≥ 18 years of age) who underwent multiplex RT-PCR testing between January 2013 and December 2015 were identified by electronic medical records. We collected clinical data using the electronic medical record on general characteristics, co-morbidities, presenting symptoms, lower respiratory complications, requirement of supplemental oxygen therapy and/or ventilatory support, hospitalization duration, and all-cause mortality. Chest radiography was performed on all patients admitted to the hospital, and radiographic interpretation was performed for all cases by radiologists. Chest CT scan was also taken 1327 (92.9%) cases among all CAP. Additional laboratory investigations were performed based on the results of a routine blood test. Sputum samples were collected for bacterial culture preparation at admission and during hospitalization. Blood cultures were also performed when indicated. Pneumonia severity index (PSI) consisted four parts, demographics including age and sex, co-morbidity, physical examination, and laboratory findings [3]. PSI score was collected every admitted patient. We contacted patients or their families by phone to identify survival and clinical information if the patients were not followed up regularly.

### Statistical analysis

Baseline characteristics (including age, sex, residency in a long-term care facility, comorbidities, presenting symptoms, and complications) at date of admission for cases and control patients were summarized using descriptive statistics, such as proportion and means (standard deviation, SD). A Chi-squared test was applied for comparison between categorical variables, and two-tailed t-tests, for comparison between continuous variables.

The univariate and multivariate Cox regression models were used to evaluate the risk of death from CAP. We analyzed survival curves for virus detected and virus not detected subjects by the Kaplan–Meier method and compared them using the log-rank test. Multivariate Cox regression was performed with each detected virus as a variable to analyze the difference in 30-day mortality according to the type of viruses. *P* values <0.05 were considered statistically significant. All statistical analyses were performed using SPSS V.21.0 (IBM, Armonk, NY).

## Results

### Clinical characteristics

Among 1,434 adult patients diagnosed and hospitalized for CAP, 261 (18%) were found to have a viral etiology and the remaining 1,173 (82%) had no virus detected. Table 1 shows the basic characteristics and outcomes of CAP patients. The mean age of the group of patients with detection of virus was 68.5 years and the mean age of the group of patients with no detection of virus was 68.3 years. Except for systolic blood pressure, there were no significant differences between body temperature, pulse rate, and respiration rate of virus-detected and virus not-detected patients with CAP. Laboratory findings did not significantly differ between virus-detected and virus non-detected CAP patients. Bacterial pathogens were detected in patient sputum at rates of 24.9% and 23.5% in both virus-detected and virus non-detected CAP patients, respectively, and the detection of bacteria in blood was almost the same between the two groups, 9.9% vs 10.0%, respectively. Measurement outcomes, such as PSI score, 30-day mortality, and 60-day mortality were not significantly different.

**Table 1:** Baseline characteristics.

Variables	Virus detected (N = 261)	Virus not detected (N = 1173)	P value
**Demographic factor**			
Male, n (%)	150 (57.4)	739 (63.0)	0.09
Age (years) mean (SD)	68.5 (14.3)	68.3 (13.9)	0.78
Age < 50 yr (%)	27 (10.3)	117 (9.9)	0.89
Resident of long-term care facilities, n (%)	21 (8.0)	92 (7.8)	0.91
**Coexisting conditions**			
Malignancy, n (%)	45 (17.2)	177 (15.0)	0.38
Congestive heart failure, n (%)	29 (11.1)	105 (8.9)	0.27
Cerebrovascular accident, n (%)	39 (14.9)	171 (14.5)	0.88
Chronic kidney disease, n (%)	40 (15.3)	149 (12.7)	0.25
Diabetes, n (%)	71 (27.2)	312 (26.5)	0.84
Liver disease, n (%)	16 (6.1)	105 (8.9)	0.13
Chronic obstructive pulmonary disease, n (%)	26 (10)	89 (7.6)	0.20
Asthma, n (%)	17 (6.5)	67 (5.7)	0.61
**Physical-examination findings**			
Altered mental status	40 (15.3)	194 (16.5)	0.63
Pulse > = 125/min	28 (10.7)	131 (11.2)	0.83
Respiratory rate > = 30/min	24 (9.2)	106 (9.0)	0.93
Systolic blood pressure < 90 mm Hg	7 (2.7)	70 (6.0)	0.03
Temperature < 35°C or > = 40°C, n (%)	3 (1.1)	17 (1.4)	0.70
**Laboratory findings**			
Blood urea nitrogen > 30 mg/dl	70 (26.8)	298 (25.4)	0.63
Glucose > = 250 mg/dl	24 (9.2)	94 (9.0)	0.53
Hematocrit < 30%	71 (27.2)	320 (27.0)	0.95
Sodium < 130 mmol/liter			
Hypoxemia (PaO2 < 60 mm Hg or O2 sat < 90%)	208 (79.7)	910 (77.6)	0.45
Arterial pH <7.35	31 (11.9)	136 (11.6)	0.89
Bacterial pathogen detected in sputum, n (%)	65 (24.9)	276 (23.5)	0.63
Bacterial pathogen detected in blood, n (%)	26 (9.9)	118 (10.0)	0.96
**PSI score, mean (SD)**	110.2 (33.0)	110.1 (35.2)	0.95
**Radiologic findings**			
Bilateral Chest CT haziness, n (%)	111 (42.5)	472 (40.2)	0.43
**Outcomes**			
Thirty-day all-cause mortality (%)	53 (20.3)	263 (22.4)	0.45
Sixty-day all-cause mortality (%)	71 (27.2)	303 (25.8)	0.64

### Mortality rate based on PSI score

The 30-day mortality rate analyzed according to the PSI score (Table 2). Mortality rate increased with an increasing PSI score in both groups. In the virus-detected CAP group, mortality began at the PSI class III (PSI 71–90). In contrast, class III CAP patients with no detected virus were found to have a mortality rate of 11%. There were no significant differences in mortality for either group outside of PSI Class III.

**Table 2:** Thirty-day mortality based on pneumonia severity index between virus detected CAP and virus non-detected CAP.

PSI score	Virus detected CAP (n, %)	30-day death (n, %)	Virus not-detected CAP (n, %)	30-day death (n, %)	P value
< 50	9 (3.5)	0 (0.0)	33 (2.8)	1 (3.0)	0.29
51–70	22 (8.0)	0 (0.0)	106 (9.0)	4 (3.7)	0.35
71–90	37 (14.1)	0 (0.0)	224 (19.1)	24 (10.7)	0.03
91–130	127 (48.7)	25 (19.7)	485 (41.4)	105 (21.6)	0.63
>130	67 (25.7)	28 (41.8)	325 (27.7)	129 (39.7)	0.75
Total	261 (100)	53 (20.3)	1173 (100)	263 (22.4)	0.45

**Fig 2:**
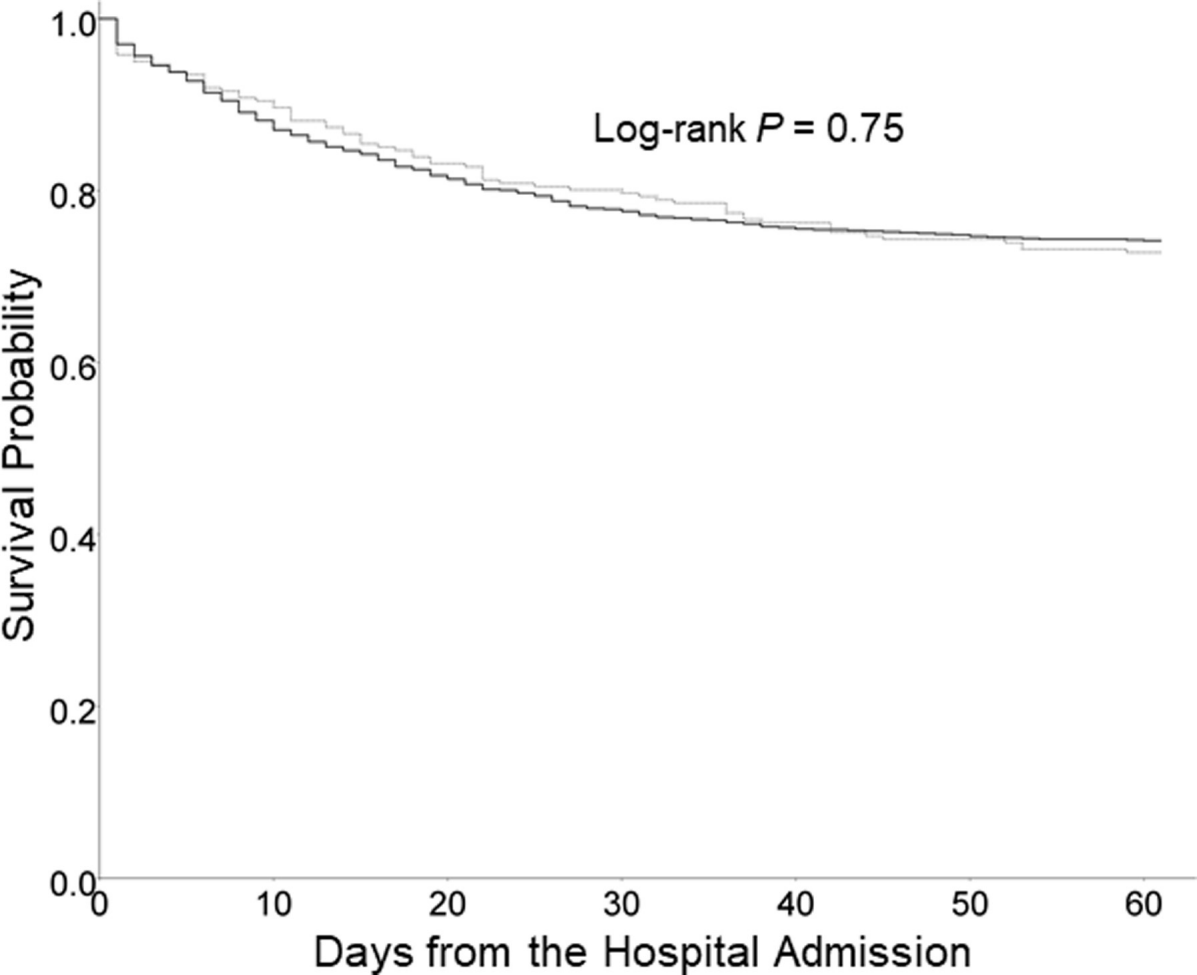
Kaplan-Meier survival curves of 1,434 adults hospitalized for community acquired pneumonia with virus detected (N = 261, dotted line) and virus not detected (N = 1,173, black line). The crude 30-day mortality of virus-detected and virus not detected pneumonia was 27.2% and 25.8%, respectively.

Multivariate analyses were performed to determine whether the detection of virus in CAP patients affects the mortality rate at 30 days. PSI classes and virus detection were used as independent variables for multivariate analyses. The presence of viruses did not affect prognosis and PSI classes had a significant effect on mortality at 30 days. PSI classes was observed as a significant variable associated with mortality at 30 days. In the PSI class V, the mortality rate was 27 times that of the PSI class I (Table 3). PSI was significantly associated with mortality adjusted for respiratory virus detection (hazard ratio = 1.024, 95% confidence interval = 1.020–1.028). The crude 30-day mortality of virus-detected and virus not-detected CAP was 27.2% and 25.8%, respectively (P = 0.75, Fig 2).

**Table 3:** Multivariate Cox regression analyses for 30-day all-cause mortality in community-acquired pneumonia.

Variables	Cases		HR	95% CI	*P* value
PSI	Death	Alive			
<50 (reference)	1	42		-	
51–70	4	128	1.32	0.14–12.18	0.80
71–90	24	261	4.11	0.54–31.21	0.17
91–130	130	612	11.06	1.50–81.13	0.01
>130	157	392	27.25	3.71–200.15	<0.01
Virus detected	53	208	0.86	0.60–1.21	0.85

PSI, pneumonia severity index; HR, hazard ratio; CI, confidence intervals

### Effects of viruses type on mortality

**Fig 3:**
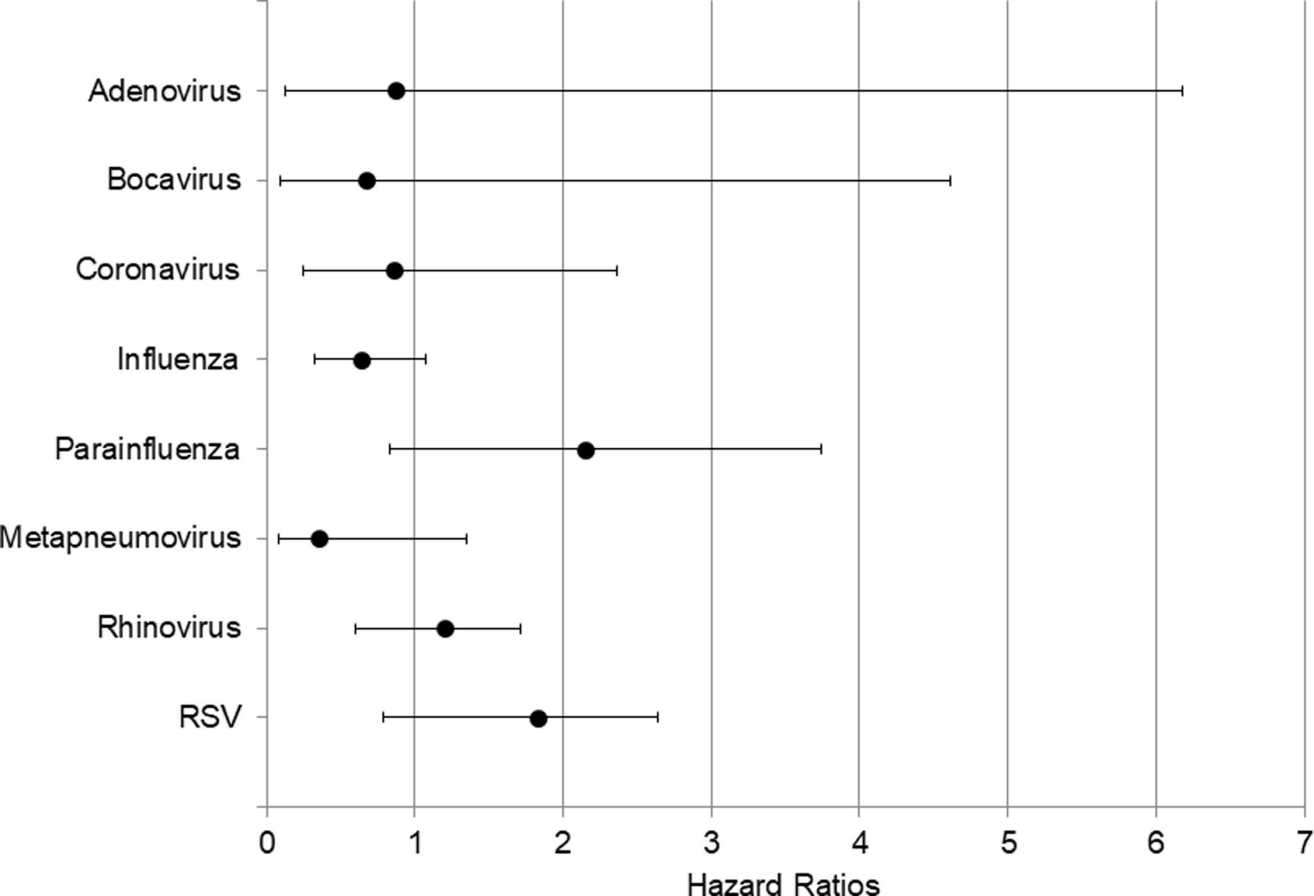
Forest plot of the hazard ratios with confidence intervals for 30-day all-cause mortality in relation to virus types in patients with community-acquired pneumonia compared with virus not detected.

Respiratory viruses were detected in 261 (18.2%) patients with community-acquired pneumonia. Two types of respiratory viruses were detected in 7 cases. Of the 254 cases detected with only one virus, 62 were influenza A, 18 were influenza B, 65 were rhinovirus, 35 were respiratory syncytial virus, 25 were metapneumovirus, 20 were parainfluenza, 17 were coronavirus, 7 were bocavirus, and 5 were adenovirus. Among 7 co-infected patients; two had bocavirus and parainfluenza, one had bocavirus and influenza A, one had bocavirus and influenza B, one had RSV and rhinovirus, one and RSV and influenza A, one had influenza A and B. The mortality rate at 30 days according to the type of virus was analyzed. Seven patients with two or more viruses were excluded from the analysis. Virus type did not have a significant effect on 30-day all-cause mortality in CAP (Fig 3).

### Bacterial pathogens

A sputum specimen from 936 (65.2%) and 1333 (92.9%) of the blood-culture specimens were obtained. A pathogen in sputum was detected in 65 (24.9%) patients among 261 virus detected CAP: gram-positive pathogens were detected in 31 patients (11.9%), and gram-negative pathogens in 34 (13.0%). A pathogen in sputum was detected in 276 (24.9%) patients among 1,173 virus not detected CAP: gram- positive pathogens were detected in 112 patients (9.5%), and gram-negative pathogens in 164 (13.9%). A pathogen in blood was detected in 26 (9.9%) patients among 261 virus detected CAP: gram-positive pathogens were detected in 12 patients (4.5%), and gram-negative pathogens in 14 (5.3%). A pathogen in blood was detected in 118 (10.0%) patients among 1,173 virus not detected CAP: gram-positive pathogens were detected in 62 patients (5.2%), and gram-negative pathogens in 56 (4.7%). Regardless of respiratory virus infection, S. aureus was most common isolated pathogen either sputum and blood (Table 4).

**Table 4:** Number and percentage of specific bacterial pathogens detected adults with community-acquired pneumonia by sample type and virus detection.

	Sputum		Blood	
Pathogen	Virus Detected (n = 261)	Virus not detected (n = 1173)	Virus Detected (n = 261)	Virus not detected (n = 1173)
*S*. *aureus*	19 (7.2%)	72 (6.1%)	7 (2.6%)	51 (4.3%)
*S*. *pneumoniae*	7 (2.6%)	24 (2.0%)	2 (0.7%)	11 (0.9%)
Other *Staphylococcus*	5 (1.9%)	16 (1.3%)	3 (1.1%)	28 (2.3%)
*Klebsiella* species	5 (1.9%)	56 (4.7%)	0 (0%)	10 (0.8%)
*Pseudomonas*	11 (4.2%)	22 (1.8%)	1 (0%)	2 (0.1%)
other *Enterobacteriaceae*	18 (6.8%)	86 (7.3%)	14 (5.3%)	44 (3.7%)

## Discussion

The PSI class indicates mortality, and the higher the score, the higher the mortality rate for both viral and non-viral CAP (Table 2). The PSI was significantly associated with mortality and adjusted for respiratory virus detection (Table 3). This study showed that the presence of virus in adult CAP patients did not affect the mortality rate compared to non-viral CAP, and is similar to previous studies.[7, 8, 11, 15].

The virus-detected CAP was significantly lower in the PSI class III group than in the virus non-detected CAP. These results suggest that viral infection has negligible effect on prognosis when co-morbidities are small or clinical features are not severe. Although there is no significant difference, our hypothesis is supported by the absence of mortality in the virus-detected CAP group, which meets the PSI class I to III (Table 2). Patient symptoms, physical exam findings, and presence of co-morbidity were suspected to have a more significant impact on prognosis than etiology of the CAP.

Some viral infections have been reported to be associated with prognosis in critically ill patients [16–20], but this study showed the type of viruses did not have a significant effect on the prognosis in patients with CAP. However, the hazard ratio for parainfluenza and RSV virus infections were increased to 2.15 (95% CI, 0.83–3.74) and 1.83 (95% CI, 0.79–2.64) compared with non-viral CAP, respectively (Fig 3). This result was similar to previous reports comparing the prognosis of RSV and influenza infection [21, 22].

As in the Spanish flu, pneumonia due to secondary bacterial infections has been identified as a major cause of death [23]. After infection with influenza virus, the frequency of secondary bacterial infections are high [24] and secondary infections by Gram-positive bacteria increase [25, 26]. Due to viral endothelial cell damage and other mechanisms, there is increased concern for bacteremia after viral infection [27–29]. Therefore, it can be assumed that in the setting of respiratory virus infection, prognosis might be affected by the 2ndary bacterial infection. The rate of positive bacterial respiratory cultures for patients with virus-detected and virus non-detected CAP remained the same. In this study, 25% of sputum positive bacterial cultures occurred in patients with virus-detected CAP, and was similar or slightly higher than in previous studies.[30, 31] The frequency of blood stream bacterial infection was 10%, and there was no bacteremia rate differences between virus-detected and virus non-detected CAP. Our study suggests that antibiotics could be considered even if viral infection is suspected in CAP.

Among CAP patients, approximately 18% were found to have a virus, and the detection rate was similar or slightly lower than that in other studies [1, 7, 32]. The frequency of detected respiratory viruses were Rhinovirus, Influenza A, and RSV in the order of 24.6%, 24.2%, and 13.8%, respectively. These results are similar to other large-scale studies [6].

It is known that the frequency of viral CAP increases as age lowers such as children. [33, 34] In this study, the mean age of the virus-detected group was 68.5 years old. In addition, the age ranges of virus-detected and non-virus-detected groups were similar. Viral respiratory infection is important in an aging society [6].

## Limitations

This study has several limitations. First, the absence of a virus in the nasopharynx does not exclude the presence of virus in the lower respiratory tract. Thus, the results may underestimate the viral etiology of lower respiratory tract infections. Second, data were retrospectively collected. Missing data and inadequate documentation such as medication start time may have resulted in study analysis bias. Third, there is a need to validate the results with a larger number of hospitals because of limited number of patients resulting in a small sample size.

## Conclusions

The mortality rate of CAP was significantly increased according to the PSI class for both virus-detected and virus non-detected groups. PSI class is a crucial factor for assessing the prognosis of patients with CAP, regardless of respiratory virus detection.

## Supporting information

S1 File The dataset for this study. (XLSX)
